# Late-Onset Immune-Related Adverse Events in Patients with Advanced Melanoma: The LATENT Study

**DOI:** 10.3390/cancers17152461

**Published:** 2025-07-25

**Authors:** Javier Pozas, Sowmya Cheruvu, Poorni Priya Jaganathan, Priya Ganesan, Arjun Modi, James Larkin, Laura Cossar, Anna Olsson-Brown, Alexandra Johnson, Nicholas Garbutt, Rebecca Lee, James Jones, Aislinn Macklin-Doherty, Kate Young, LATENT Study Investigators

**Affiliations:** 1 Department of Medical Oncology, The Royal Marsden Hospital NHS Foundation Trust, London SW3 6JJ, UKpoorni.jaganathan@rmh.nhs.uk (P.P.J.); priya.ganesan@nhs.net (P.G.); arjun.modi@rmh.nhs.uk (A.M.); aislinn.macklin-doherty@rmh.nhs.uk (A.M.-D.); 2 Department of Medical Oncology, University College London Hospitals NHS Trust, London NW1 2PB, UK; 3 Department of Medical Oncology, The Clatterbridge Cancer Centre NHS Foundation Trust, Liverpool CH63 4JY, UKnicholas.garbutt@nhs.net (N.G.); 4 Department of Medical Oncology, The Christie NHS Foundation Trust, Manchester M20 4BX, UKjames.jones21@nhs.net (J.J.)

**Keywords:** advanced melanoma, immune checkpoint inhibitors, late-onset toxicities

## Abstract

Background/Objectives: Immune checkpoint inhibitors have significantly transformed the treatment paradigm of advanced melanoma, leading to substantial improvements in survival outcomes. However, this therapeutic success is accompanied by a spectrum of treatment-related adverse events, some of which are increasingly recognised as enduring and non-reversible. Whilst early-onset immune-related toxicities have been well characterized, late-onset toxicities, often emerging in patients with long-term disease control, remain understudied and are frequently overlooked. Methods: To address this knowledge gap, we conducted a retrospective multicentre study in three UK tertiary referral centres, exploring immune-related adverse events in 246 patients with melanoma who received immune checkpoint inhibitors in the advanced setting. We defined late-onset immune-related adverse events as those occurring at least 3 months after the last cycle of immune checkpoint inhibitors. Results: Although most patients experienced early-onset toxicity, almost 15% of patients developed late-onset immune-related adverse events, including skin rash, colitis, hepatitis, and arthritis, among others. These were often challenging to manage and necessitated the use of systemic steroids. Up to 2% of patients presented ultra-late-onset toxicities, defined as those events occurring at least 12 months after treatment completion. Conclusions: This study provides valuable insights into the characteristics of late-onset immune-related adverse events. To further advance our understanding of these late-onset toxicities, dedicated prospective studies are needed to assess risk factors associated with their development and their impact on quality of life. Additionally, translational research focused on finding predictive biomarkers is essential to identify patients at a higher risk of developing delayed adverse events and to understand how best to manage them.

## Simple Summary

Immune checkpoint inhibitors (ICIs) have significantly improved survival in patients with advanced melanoma. Over the past decade, the early detection and management of immune-related adverse events (irAEs) during ICI treatment have markedly advanced. Although there is increasing awareness concerning late-onset irAEs, they remain a diagnostic and therapeutic challenge. In our study, approximately 15% of patients developed late-onset irAEs, with almost all patients (92%) having an early-onset irAE, of which 22% were severe and over half required treatment with corticosteroids and/or additional immunosuppressive therapies. These findings underscore the critical need for clinicians to maintain the vigilant, long-term monitoring of patients receiving ICIs, even after treatment discontinuation.

## 1. Introduction

The advent of immune checkpoint inhibitors (ICIs) has revolutionized the treatment of advanced melanoma, with unprecedented improvements in survival rates and durable responses in a high percentage of patients. These therapies, including cytotoxic T-lymphocyte antigen-4 (CTLA-4) inhibitors (e.g., ipilimumab) and programmed cell death-1 (PD-1) inhibitors (e.g., nivolumab and pembrolizumab), leverage the host immune system to target and kill tumour cells [1,2,3,4,5,6]. While their introduction has been a paradigm shift in melanoma management, ICIs are increasingly used across multiple tumour types. As their use continues to grow and has expanded into the adjuvant and even neoadjuvant settings, understanding immune-related adverse events (irAEs)—including late-onset toxicities—has become essential for optimizing long-term patient care.

ICIs function by restoring or enhancing T cell activity against cancer cells, but this mechanism also exposes patients to irAEs. These toxicities are the result of immune-mediated inflammation of normal tissues and can affect any organ, most commonly the skin, gastrointestinal tract, liver, and endocrine glands [7].

Despite the limited understanding of the pathophysiology of irAEs, it is believed to involve the hyperactivation of the immune system, a loss of self-tolerance, and the cross-reactivity of T cells with antigens expressed in both tumours and normal tissues. Genetic predisposition, gut microbiome composition, and tumour-related factors may also modulate the risk and severity of irAEs [8,9].

IrAEs are most frequent during the first three months of treatment but may occur at any time during treatment or even months after therapy is discontinued. Late-onset toxicities represent an increasingly recognized subset of irAEs [10], which have emerged as follow-up time and experience with ICIs have increased. One of the most critical features of delayed irAEs is their heterogeneity, both in clinical presentation and natural history. There has been some discussion about how best to define and capture different types of late-onset irAEs. Acute or early-onset irAEs have been described by some as those that occur in the first 12 months of treatment and delayed or late-onset irAEs as those that occur after 12 months of treatment. Based on the recent consensus published by the Society for Immunotherapy of Cancer (SITC), we have defined delayed or late-onset irAEs as those that are diagnosed more than 3 months after the discontinuation of immunotherapy. The delayed presentation complicates diagnosis and management, as the time interval between exposure to ICI and toxicity may confound the causal link [11].

Endocrinopathies, such as hypothyroidism, adrenal insufficiency, and diabetes mellitus, are among the most frequently observed late-onset toxicities, often presenting with insidious symptoms that may be mistaken for other conditions. Other late-onset toxicities include myocarditis, pneumonitis, nephritis, and neurological syndromes, which can be life-threatening if not diagnosed and treated early.

Acute toxicities have been well characterized in clinical trials and large retrospective series. However, only a handful of studies have focused on the characterization of late-onset irAEs. The purpose of this study was to investigate the occurrence and characteristics of these late-onset irAEs in patients with advanced melanoma treated with ICIs.

## 2. Materials and Methods

With approval from the National Research Ethics Committee (24/WA/0225), we conducted a retrospective analysis involving data collected from hospital records from three tertiary cancer care centres across the UK. This study included patients with unresectable stage III or stage IV melanoma treated between 2008 and 2021 who achieved a prolonged response to ICIs lasting at least three years, defined as the time from date of first cycle of an ICI until date of last clinical review with no evidence of disease progression. Eligible treatments encompassed single-agent anti-PD-1 or anti-CTLA-4 therapies, as well as combination regimens involving both agents. While prior systemic therapies, including chemotherapy and targeted therapies, were allowed, ICIs were required to be the most recent line of treatment before inclusion in the study.

The incidence of irAEs was captured from diagnoses made and recorded within hospital records of specialist melanoma clinics and calculated as a proportion of the total number of patients included in this study. An irAE was defined as late-onset if it developed at least 3 months after the last cycle of treatment as per the SITC consensus. irAEs were categorised by the affected organ system according to the Common Terminology Criteria for Adverse Events (CTCAE) version 5. This was determined by the reviewing clinician at the time of review or by the description of severity of symptoms by a specialty trained oncologist at the time of data collection. Data were collected on baseline disease characteristics, the severity of irAEs (graded 1 to 5), time to onset, and the need for organ-specific specialist referral. The use of systemic steroids and other immunosuppressive agents for the management of irAEs was also recorded. We have adopted the term “ultra-late-onset irAEs” to describe those irAEs that emerged 12 months after the last cycle of treatment with an ICI.

Descriptive statistics were used to summarize patients’ characteristics and adverse events. Statistical analyses were performed using Python 3. Categorical variables were analysed using the Pearson’s Χ^2^ test to determine the association between baseline characteristics and the incidence of late-onset irAEs. Continuous variables were compared using one-way analysis of variance (ANOVA). A *p*-value of <0.05 was considered statistically significant.

## 3. Results

### 3.1. General Characteristics

A total of 246 patients were included across the three sites. General characteristics are summarized in Table 1. There was a male preponderance (ratio male/female 1.44), and the median age at diagnosis of advanced disease was 63 years. As expected, the majority of patients had cutaneous melanoma and approximately 30% of patients harboured a *BRAF* mutation. Approximately 15% and 20% of patients had CNS and liver metastases at diagnosis, respectively. The majority of patients (86.4%) received ICIs in the frontline setting. The combination of ipilimumab and nivolumab accounted for the majority of first-line treatment (45.1%) with 32.9% of patients receiving single-agent anti-PD-1 (Figure S1).

### 3.2. Development of irAEs in the Study Cohort

**Figure 1:**
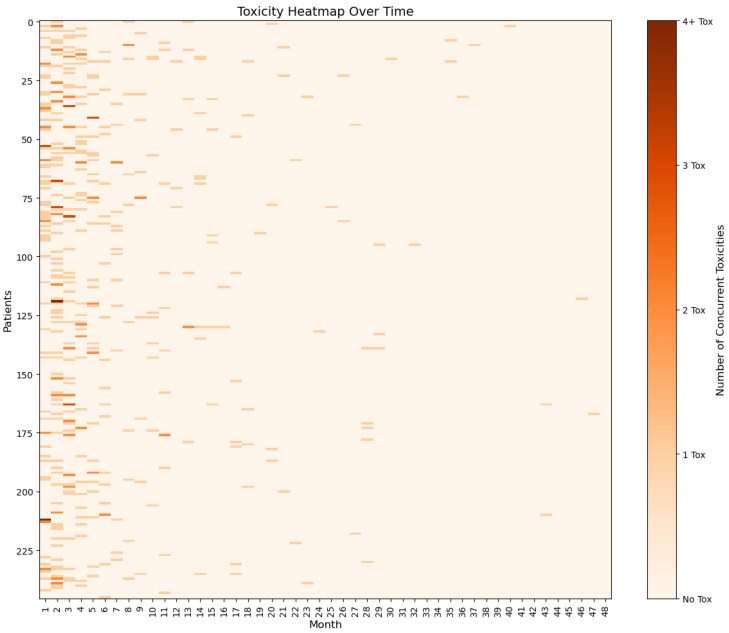
A heatmap showing the development of irAEs since the start of treatment with ICIs. The different colour intensity represents the number of concurrent toxicities.

**Figure 2:**
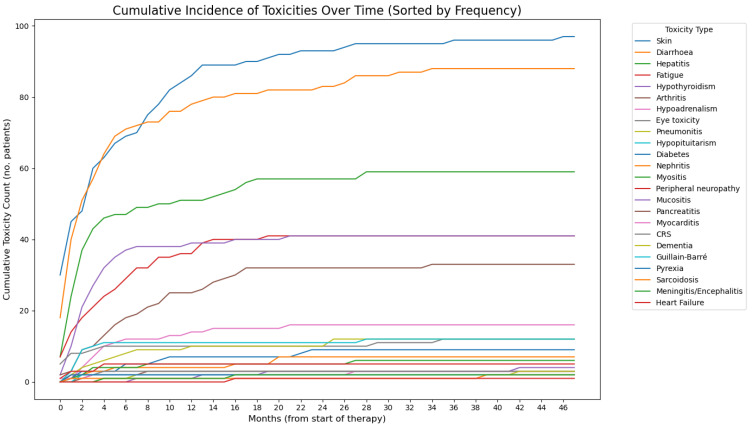
A line chart showing the incidence of irAEs over time, sorted by frequency.

Most patients (81%) developed at least one irAE and approximately 40% of patients experienced grade 3 or 4 irAEs (Table 2). The most frequent irAEs were skin rash (42%), diarrhoea (36%), hepatitis (25%), fatigue (17%), hypothyroidism (17%), and rheumatological events (14%). In the majority of cases, irAEs developed within the first 3 months of therapy with ICIs (Figure 1 and Figure 2). The median time of onset was between two and three months for rash, diarrhoea, hepatitis, fatigue, and hypothyroidism. Rheumatological adverse events tended to occur later, with a median time of onset of 6 months. Almost 50% and 40% of patients with G3/G4 colitis and rash were referred for specialist care, respectively. Conversely, none of the patients with grade 1 or grade 2 adverse events required specialist referral, and these irAEs were managed by the primary oncology team.

Approximately 60% of patients who developed irAEs required systemic steroids (Table 3 and Table S1). A significant proportion of patients with colitis warranted intravenous steroids, and some also required the use of additional immunosuppressive agents, most frequently infliximab and vedolizumab. IrAEs affecting the central nervous system, such as meningitis/encephalitis or Guillain–Barré syndrome, were rare (<1%) but always necessitated the use of high-dose IV steroids and/or other immunosuppressants.

### 3.3. Late-Onset irAEs

**Figure 3:**
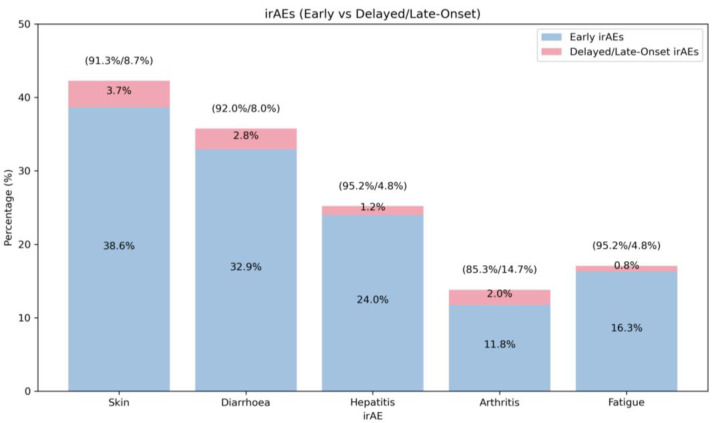
A stacked bar chart illustrating the incidence of most frequent irAEs. The percentage values in brackets above each bar represent the individual contributions of early-onset and late-onset irAEs to the total incidence of each irAE.

A total of 36 patients (14.6%) developed late-onset irAEs (Table 4), starting more than 3 months after immunotherapy discontinuation. As illustrated in Figure 3, late-onset irAEs contributed to less than 10% of the total number of events in most cases, except for arthritis, where it almost reached 15%. There was no statistical association between delayed toxicity and clinicopathological variables, including age, gender, baseline autoimmune conditions, ethnicity, histological subtype, *BRAF* or *NRAS* mutation, presence of brain or liver metastases at diagnosis, high LDH levels at diagnosis, and choice of first-line therapy.

The vast majority of patients with late-onset irAEs (33/36) had previously developed at least one irAE. Almost 19% (24/128) of patients who received ipilimumab as their last line of therapy (single-agent or in combination with nivolumab) developed late-onset irAEs, compared to 10% (12/118) of patients who were treated with single-agent anti-PD-1. This association, however, did not reach statistical significance (*p* = 0.08).

A total of 13 (36.1%), 15 (41.7%), and 8 (22.2%) patients had grade 1, grade 2, and grade 3 late-onset toxicities, respectively. Half of the patients (50%) needed systemic steroids for the management of the irAEs, and two patients required other immunosuppressants.

### 3.4. Ultra-Late-Onset irAEs

Five patients developed six ultra-late-onset irAEs, occurring 12 months or more after stopping immunotherapy treatment. This contrasts with almost 15% of patients who developed late-onset toxicities. These cases are summarized in Table 4 and Table 5.

The first patient was a 30-year-old male at the time of diagnosis, who developed nodal disease 3 years after the resection of localised cutaneous melanoma. This was resected, but a few months later, a restaging scan picked up solitary brain metastasis. He underwent the excision of the lesion followed by post-operative radiotherapy, and was then commenced on 3 mg/kg ipilimumab and 1 mg/kg nivolumab. He completed four cycles of combination and 2 years of single-agent nivolumab thereafter as per standard guidelines. He tolerated treatment well and only experienced grade 1 skin toxicity, grade 1 hepatitis, and grade 2 hypothyroidism during the combination part of the treatment. However, around 15 months after the completion of ICI therapy, a surveillance scan revealed enlarged mediastinal lymphadenopathies, for which the patient was asymptomatic. He was referred to a respiratory physician who organized an EBUS-guided biopsy. This revealed non-necrotizing granulomas, in keeping with a sarcoid-like reaction to the previous treatment with ICIs. Given the lack of symptoms, systemic steroids were not initiated, and the appearances slowly improved over the following surveillance scans.

Patient number 2 was a 67-year-old male who presented with the mucosal melanoma of the nasal cavity, which was initially excised. He then experienced multiple local recurrences over the following two decades that were managed with radiotherapy and surgery, but eventually developed lung metastasis 20 years after the initial diagnosis. He was started on 3 mg/kg single-agent nivolumab, which was discontinued after cycle 9 due to ICI-induced diarrhoea that required hospital admission and treatment with high-dose intravenous steroids. Around 17 months after the last cycle of treatment, he presented with visual disturbances in the lower right eye’s visual field. An initial assessment by his local optician revealed the marked swelling of the optic disc. A CT scan ruled out brain metastases, and he was referred to our specialized ophthalmologist who confirmed the diagnosis of papillitis, likely in the context of a previous exposure to ICIs. His symptoms resolved after a course of topical and systemic steroids.

The third patient was a 58-year-old female with a background of rheumatoid arthritis who had stage II cutaneous melanoma resection. Four years later, she developed widespread metastatic disease involving the lymph nodes, liver, and lungs. She received treatment with 3 mg/kg ipilimumab and 1 mg/kg nivolumab. Treatment was discontinued due to cumulative toxicity and maintained clinical response. Approximately 12 months after the last cycle of nivolumab, the patient developed grade 2 hepatitis and grade 1 pneumonitis. Extensive diagnostic work-up was carried out, including functional imaging, blood test, serology screen, and bronchoscopy, ruling out other common causes. The liver function improved after a short course of steroids, and no intervention was required for the pneumonitis.

The fourth patient was a 71-year-old female who was diagnosed with stage IV anal mucosal melanoma. She received three cycles of 3 mg/kg ipilimumab and 1 mg/kg nivolumab, after which she experienced grade 3 ICI-induced colitis, and the treatment was discontinued. Almost 4 years after the last cycle of treatment, she developed a maculopapular rash with small areas of ulceration, in keeping with ICI toxicity given the lack of other causative agents. It resolved with oral and topical steroids, anti-histamines, and emollients.

The final patient was a 75-year-old male who presented initially with stage III melanoma. He developed lung metastases shortly after and was treated with single-agent pembrolizumab. Treatment had to be discontinued after four cycles due to grade 3 immune-related nephritis, but had an excellent response to treatment and never required further therapies for his melanoma. Over three years after the last cycle of pembrolizumab, he developed memory problems. After thorough investigation including brain functional imaging, extensive blood work, and neuropsychological testing, the local specialists concluded that this could be attributed to his previous ICI treatment.

## 4. Discussion

The findings from this multicentre UK-based study provide real-world insights into the occurrence and clinical implications of late-onset irAEs in patients with advanced melanoma treated with ICIs. Approximately 15% of patients developed late-onset irAEs, of which 22% were graded as severe and half of them needed systemic immunosuppressant treatment.

As a retrospective real-world study, there is positive selection for patients in terms of response to treatment and potential impacts on profile and types of irAEs that may occur. The proportion of our patients being diagnosed with any grade (81%) or severe (40%) irAEs is consistent with that reported in many studies involving both PD1-/PD-L1 and CTLA-4 ICI treatments, suggesting the comprehensive capture of these events through study centre clinical records [4,12].

The incidence of late-onset irAEs was relatively high (approximately 15%), above the 5.7% reported by Noseda et al. in a large pharmacovigilance study, using the same timeline for late-onset irAEs [13]. The lower incidence of irAEs may have different causes. Their cohort included patients with multiple cancer types, many of which are inherently less sensitive to ICIs than melanoma. These cancers may harbour a more immunosuppressive tumour microenvironment, reducing the likelihood of irAEs. Also, non-responders were included, which introduces a bias and dilutes the irAE rate. The spectrum of affected organs in our study was quite diverse, and systemic steroids were required in many cases, particularly in colitis, hepatitis, and arthritis. These findings are in line with previous retrospective studies [11,14].

The lack of a standardised definition for late-onset irAEs complicates direct comparisons across studies [15,16]. While some studies define late-onset irAEs as those occurring beyond one year after treatment initiation, others categorise them based on their emergence after therapy completion [11,14,17,18]. The variability in definitions highlights the need for a uniform classification system. In an effort to standardise definitions, the SITC published a consensus defining delayed or late-onset irAEs as those occurring at least three months after the discontinuation of immunotherapy [10]. While this represents an important first step, establishing a widely accepted and prospectively validated threshold for late-onset irAEs would improve the consistency of data interpretation and facilitate more effective patient monitoring.

The rate of late-onset irAEs was higher in patients treated with anti-CTLA-4 as single-agent or in combination with anti-PD-1. This is consistent with the results of a large real-world analysis conducted by Yang and colleagues, which involved more than 100,000 patients treated with ICIs [17]. In our cohort, most patients who developed late-onset irAEs had previously experienced at least one irAE.

Unlike early-onset irAEs, which often present with overt and rapidly progressive symptoms, late-onset toxicities can present insidiously. This can lead to delayed recognition and misattribution to other medical conditions, particularly in patients who are no longer undergoing frequent oncological follow-up [19,20]. In our study, the delayed onset of infrequent irAEs, such as sarcoidosis or papillitis, highlights the need for a high clinical suspicion when evaluating patients with a history of ICI exposure. Given that some of these toxicities can mimic metastatic progression (e.g., sarcoidosis-like reaction), careful clinical, radiological, and histopathological assessments are warranted to avoid any unnecessary interventions [21,22].

One of the strengths of our study is that it exclusively included patients with melanoma who responded to immunotherapy and did not require subsequent lines of treatment. This prevented the impact that other systemic therapies could have had on toxicity attribution, as well as the confounding effect of co-morbidities, such as the high rates of pneumonitis in patients with lung cancer.

Our study also focused on ultra-late-onset irAEs. Evidence on ultra-late irAEs remains scarce, and, to the best of our knowledge, this is one of the first studies to systematically investigate these events in patients who continue to respond to immunotherapy. These events hold special relevance, as most patients at this stage undergo less intensive monitoring. Current clinical guidelines recommend follow-up every 3 to 6 months after treatment discontinuation, making the early detection and management of irAEs challenging [23]. Nakai et al. reported two cases of irAEs occurring more than one year after stopping treatment. While both patients responded to corticosteroids, they had recently initiated tyrosine kinase inhibitor (TKI) therapy, confounding the causal relationship between ICIs and the observed irAEs [24].

Several mechanisms have been proposed to explain the origin of irAEs, including a loss of self-tolerance, the hyperactivation of autoreactive T and B cells, cytokine dysregulation, the enrichment of the microbiome with bacterial species that induce toxicities, and tissue-specific immune responses [25]. The molecular pathways of delayed irAEs remain largely unknown, and we lack useful molecular biomarkers to predict their occurrence. Long-term exposure to ICIs could allow autoreactive T cells to gradually accumulate, leading to delayed toxicities. Another possible mechanism could be epitope spreading, in which after the initial reactivity to tumour-associated antigens, the immune system starts reacting to self-antigens. Finally, the prolonged disturbances of the Treg compartment and cytokine dysregulation may initially remain below the clinical threshold but eventually trigger overt tissue damage [26,27,28].

Our study has several limitations. The retrospective nature of data collection introduces inherent biases as we have discussed, including the underreporting of late toxicities, the misattribution of clinical diagnoses to late-onset irAEs, and inconsistencies in clinical documentation with respect to the grading severity of toxicities and dates of diagnosis, which would not occur in a prospective clinical trial. However, the fact that the proportion affected by irAEs concurs with clinical studies is very reassuring as it indicates that there is minimal misclassification. Additionally, it can be especially challenging to definitively attribute certain clinical conditions, such as memory loss, to irAEs. Further specific research is required to understand the risks and mechanisms of cognitive impairment associated with ICIs. Subtle or subclinical toxicities, such as grumbling myocarditis or infertility, may have been underrecognized due to the reliance on patient/clinician-reported outcomes and the absence of systematic long-term follow-up. However, this represents a more general limitation in terms of diagnosing late-onset irAEs and is not specific to our study. Also, the profile of late-onset irAEs in our study could have been impacted by the positive selection of long-term responders, as non-responders were excluded.

Future research should focus on characterizing the biological mechanism for late-onset irAEs as distinct from acute irAEs as there is little known about them. There is also a great need to improve the sensitivity of capturing late-onset irAEs. For this purpose, incorporating prospective focussed toxicity data collection into follow-up clinics, as well as patient-reported outcome measures (PROMs), would better capture the full spectrum of late-onset toxicities, particularly more insidious onset irAEs, where patient-reported outcomes may improve the sensitivity of diagnosis, as recently suggested by Goergopoulou and colleagues [29]. PROMs could also capture relapsing/remitting toxicities, which are not severe enough to warrant treatment but still impact the patients’ quality of life. As ICIs are increasingly used across different malignancies and in earlier treatment settings, long-term follow-up will be essential to identify delayed toxicities that may otherwise go unnoticed, such as late cardiac toxicity [30]. Further studies should also aim to identify biomarkers for predicting late-onset irAEs and develop strategies for their prevention and management.

## 5. Conclusions

In summary, late and ultra-late-onset irAEs affect a small yet clinically significant subset of patients with advanced melanoma. Early detection is challenging due to less frequent follow-up and low clinical suspicion. Late-onset irAEs can be severe and often require treatment with systemic immunosuppression. We lack useful biological or clinical biomarkers to identify patients at a higher risk of developing delayed irAEs, which warrants the development of large international prospective cohorts. Similarly, translational research is essential to unravel the molecular mechanisms underlying late-onset toxicities. Until then, clinicians need to be vigilant and carefully monitor patients who have been exposed to ICIs, even after treatment discontinuation. Finally, the oncology community has the responsibility to educate patients and other healthcare professionals about the toxicity profile of ICIs.

## Figures and Tables

**Table 1:** General characteristics.

Variable		Patients (*n* = 246) (%)
Gender—no. (%)		
	Male	145 (58.9)
	Female	101 (41.1)
Age—median (range)		63 (18–92)
Melanoma subtype—no. (%)		
	Cutaneous	152 (85.9)
	Mucosal	8 (4.5)
	Acral	4 (2.3)
	Other	13 (7.3)
Mutational profile—no. (%)		
	*BRAF* mutant	79 (32.1)
	*NRAS* mutant	24 (9.8)
Extent of disease at stage IV diagnosis—no. (%)		
	Brain metastases	36 (14.6)
	Liver metastases	49 (19.9)
Number of lines—no. (%)		
	1	191 (77.6)
	2	43 (17.5)
	3	8 (3.3)
	4	4 (1.6)
First-line treatment regimens—no. (%)		
	Ipilimumab/nivolumab	111 (45.1)
	Pembrolizumab	60 (24.4)
	Ipilimumab	23 (9.3)
	Nivolumab	21 (8.5)
	Dacarbazine	8 (3.3)
	Dabrafenib/trametinib	8 (3.3)
	Melphalan	4 (1.6)
	Vemurafenib	3 (1.2)
	Cisplatin/dacarbazine	3 (1.2)
	Bevacizumab	1 (0.4)
	Temozolomide	1 (0.4)
	MEK inhibitor (clinical trial)	1 (0.4)
	Docetaxel	1 (0.4)
	Dabrafenib	1 (0.4)
Last line of treatment—no. (%)		
	Ipilimumab/nivolumab	115 (46.7)
	Pembrolizumab	87 (35.4)
	Nivolumab	31 (12.6)
	Ipilimumab	13 (5.3)

**Table 2:** Immune-related adverse events (irAEs).

Variable		Patients (*n* = 246) (%)
Any irAE—no. (%)		199 (80.9)
Most frequent irAEs (>10%)—no. (%)		
	Rash	104 (42.3)
	Diarrhoea	88 (35.8)
	Hepatitis	62 (25.2)
	Fatigue	42 (17.1)
	Hypothyroidism	41 (16.7)
	Arthritis/arthralgia	34 (13.8)
Any G3/G4 irAEs—no. (%)		97 (39.4)
Most frequent G3/4 irAEs (>5%)—no. (%)		
	Diarrhoea	43 (17.5)
	Hepatitis	36 (14.6)
	Rash	13 (5.28)
Time of onset (months)—median (range)		3 (0–46)
	Rash	3 (0–46)
	Diarrhoea	2 (0–34)
	Hepatitis	2 (0–28)
	Fatigue	3 (0–19)
	Hypothyroidism	2 (0–21)
	Arthritis/arthralgia	6 (0–34)
Referral to specialist (among patients with G3/G4 irAEs)—no. (%)		
	Rash	5 (38.5)
	Diarrhoea	20 (46.5)
	Hepatitis	8 (22.2)
	Arthritis/arthralgia	1 (20)
Median time to referral (months)—median (range)		0 (0–22)

**Table 3:** Management of irAEs.

Variable		Patients (*n* = 199)		
		Total	Oral	IV
Systemic steroids—no. (%)		122 (61.3%)	118 (59.4%)	68 (34.2%)
	Rash (n = 104)	24 (23.1%)	24 (23.1%)	5 (4.81%)
	Diarrhoea (n = 88)	66 (75%)	65 (73.9%)	48 (54.6%)
	Hepatitis (n = 62)	29 (25.2%)	24 (38.7%)	6 (9.7%)
	Arthritis (n = 34)	30 (88.2%)	30 (88.2%)	1 (2.94%)
	Pneumonitis (n = 12)	9 (75%)	9 (75%)	0
	Ocular toxicity (n = 12)	4 (33.3%)	4 (33.3%)	1 (8.3%)
	Nephritis (n = 7)	4 (57.1%)	4 (57.1%)	2 (28.6%)
	Myositis (n = 6)	4 (66.7%)	4 (66.7%)	0
	Peripheral neuropathy (n = 5)	2 (40%)	2 (40%)	2 (40%)
	Myocarditis (n = 3)	2 (66.7%)	2 (66.7%)	2 (66.7%)
	CRS (n = 3)	1 (33.3%)	1 (33.3%)	1 (33.3%)
	Pancreatitis (n = 3)	1 (33.3%)	1 (33.3%)	1 (33.3%)
	Memory loss (n = 3)	1 (33.3%)	1 (33.3%)	0
	Meningitis/encephalitis (n = 2)	2 (100%)	2 (100%)	2 (100%)
	Guillain–Barré (n = 2)	2 (100%)	2 (100%)	1 (50%)
	Sarcoidosis (n = 2)	1 (50%)	1 (50%)	0
Other immunosuppressants—no. (%)		55 (27.6%)		
	Rash (n = 104)	3 (2.9%)		
	Diarrhoea (n = 88)	29 (33%)		
	Hepatitis (n = 62)	18 (29%)		
	Arthritis (n = 34)	9 (26.5%)		
	Pneumonitis (n = 12)	1 (8.33%)		
	CRS (n = 3)	1 (33.3%)		
	Pancreatitis (n = 3)	1 (33.3%)		
	Guillain–Barré (n = 2)	1 (50%)		

CRS, cytokine release syndrome.

**Table 4:** Late-onset and ultra-late-onset immune-related adverse events (irAEs).

Variable		Value (*n* = 246) (%)
Late-onset irAEs—no. (%)		36 (14.6)
	Rash	9 (3.7)
	Diarrhoea	7 (2.8)
	Arthritis	5 (2)
	Hepatitis	3 (1.2)
	Memory loss	3 (1.2)
	Nephritis	2 (0.8)
	Hypoadrenalism	2 (0.8)
	Myositis	2 (0.8)
	Mucositis	2 (0.8)
	Fatigue	2 (0.8)
	Pneumonitis	1 (0.4)
	Sarcoidosis	1 (0.4)
	Diabetes	1 (0.4)
	Eye toxicity	1 (0.4)
	Heart failure	1 (0.4)
	Myocardial infarction	1 (0.4)
Ultra-late-onset irAEs—no. (%)		5 (2)
	Pneumonitis	1 (0.4)
	Rash	1 (0.4)
	Hepatitis	1 (0.4)
	Sarcoidosis	1 (0.4)
	Ocular toxicity	1 (0.4)
	Dementia	1 (0.4)

**Table 5:** Patients with ultra-late-onset toxicities.

Patient	Gender, Age	BRAF	Primary	Baseline LDH (U/L)	Treatment	Toxicity	Time from Last Treatment to Toxicity Presentation	Toxicity	Time from Last Treatment	Other Toxicities
1	M, 30	Mut	Cutaneous	117	Ipi/Nivo	Sarcoidosis	15 months	–	–	Hypothyroidism, hepatitis, skin rash
2	M, 67	WT	Mucosal	195	Nivolumab	Papillitis	17 months	–	–	Skin rash, diarrhoea, fatigue, diabetes
3	F, 58	WT	Cutaneous	NA	Ipi/Nivo	Hepatitis	18 months	Pneumonitis	18 months	Arthritis, hypothyroidism, hypoadrenalism
4	F, 71	WT	Mucosal	361	Ipi/Nivo	Skin rash	44 months	–	–	Diarrhoea
5	M, 75	WT	Unknown	243	Pembrolizumab	Dementia	38 months	–	–	Hypothyroidism, nephritis

## Data Availability

The datasets generated and/or analysed during this study are not publicly available due to patient confidentiality and privacy restrictions. The data contains sensitive personal health information protected under institutional ethics guidelines. Requests for limited, de-identified data may be considered upon reasonable request to the corresponding author, subject to approval by the institutional ethics committee.

## Supplementary Materials

The following supporting information can be downloaded at: https://www.mdpi.com/article/10.3390/cancers17152461/s1, Figure S1: Pie charts representing treatment distribution in first, second, third and fourth line of therapy; Table S1: Use of steroids for the management of irAEs (early vs. late-onset toxicity).

## Abbreviations

The following abbreviations are used in this manuscript:

ICI	Immune checkpoint inhibitor
CTLA-4	Cytotoxic T-lymphocyte antigen-4
PD-1	Programmed cell death-1
irAE	Immune-related adverse event
CTCAE	Common Terminology Criteria for Adverse Events
CNS	Central nervous system
TKI	Tyrosine kinase inhibitors
CRS	Cytokine release syndrome
NA	Not applicable
