# Supplementary material for: Late-Onset Immune-Related Adverse Events in Patients with Advanced Melanoma: The LATENT Study
Source: Cancers (Basel). 2025 Jul 25;17(15):2461. doi: 10.3390/cancers17152461 (PMC12346639; doi:10.3390/cancers17152461)
Supplement: Supplementary file 1 [file cancers-17-02461-s001.zip › cancers-3714443-supplementary.pdf]

Supplementary materials

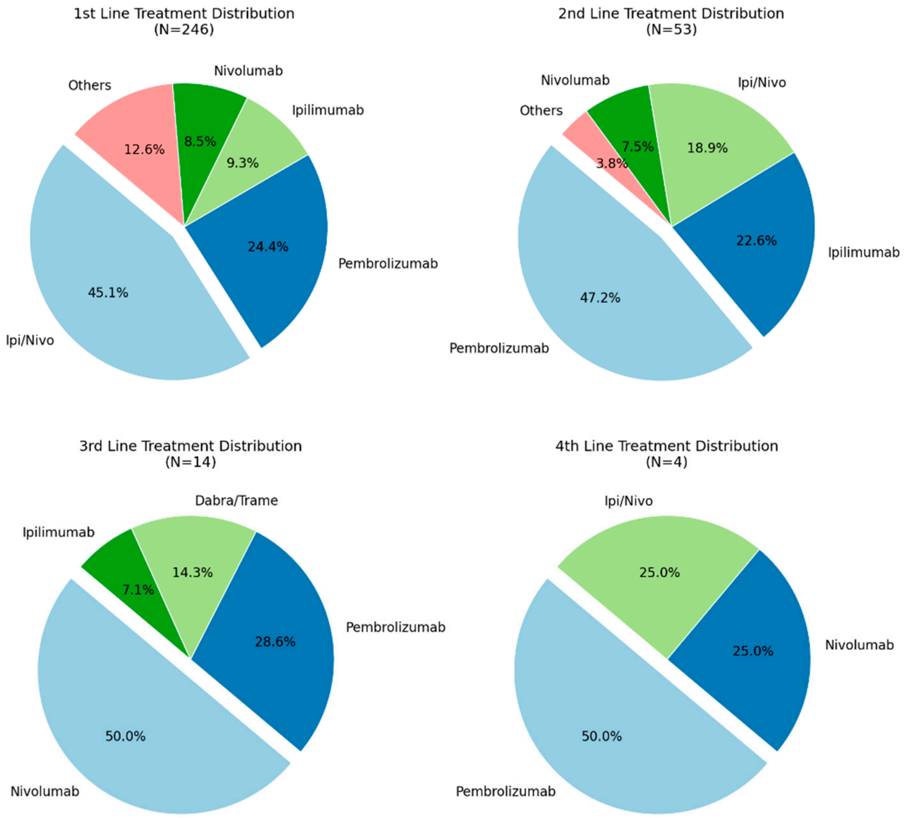

Figure S1**.** Pie charts representing treatment distribution in first, second, third and fourth line of therapy.

Table S**1.** Use of steroids for the management of irAEs (early vs late-onset toxicity).

Total number of patients with (early) and (late) onset irAEs	**Early-onset toxicity (< 3 months) n = 196**			**Late-onset toxicity (≥ 3 months) n = 36**		
	Systemic steroids (highest escalation) – no. (%)					
	Total	Oral	IV	Total	Oral	IV
Rash (n = 95), (n = 9)	20 (21.1)	16 (21.1)	4 (4.2)	4 (44.4)	3 (44.4)	1 (11.1)
Diarrhoea (n = 81), (n = 7)	60 (74.1)	15 (18.5)	45 (55.6)	6 (85.7)	3 (85.7)	3 (42.9)
Hepatitis (n = 59), (n = 3)	29 (49.2)	23 (40)	6 (10.2)	0	0	0
Arthritis (n = 29), (n = 5)	25 (86.2)	24 (82.8)	1 (3.4)	5 (100)	5 (100)	0
Pneumonitis (n = 11), (n = 1)	8 (72.7)	8 (72.7)	0	1 (100)	1 (100)	0
Eye toxicity (n = 11), (n = 1)	4 (36.4)	3 (27.3)	1 (9.1)	0	0	0
Nephritis (n = 5), (n = 2)	4 (80)	2 (40)	2 (40)	0	0	0
Myositis (n = 4), (n = 2)	3 (75)	3 (75)	0	1 (50)	1 (50)	0
Peripheral neuropathy (n = 5)	2 (40)	0	2 (40)	NA	NA	NA
Myocarditis (n = 3)	2 (66.7)	0	2 (66.7)	NA	NA	NA
CRS (n = 3)	1 (33.3)	0	1 (33.3)	NA	NA	NA
Pancreatitis (n = 3)	1 (33.3)	0	1 (33.3)	NA	NA	NA
Memory loss (n = 3)	NA	NA	NA	1 (33)	1 (33)	0
Meningitis/Encephalitis (n = 2)	2 (100)	0	2 (100)	NA	NA	NA
Guillain-Barré (n = 2)	2 (100)	1 (50)	1 (50)	NA	NA	NA
Sarcoidosis (n = 1), (n = 1)	1 (100)	1 (100)	0	0	0	0

CRS, cytokine release syndrome. NA, not applicable.
